# Editorial: Community series in trends in neuroimmunology: cross-talk between brain-resident and peripheral immune cells in both health and disease, volume II

**DOI:** 10.3389/fimmu.2025.1644278

**Published:** 2025-07-03

**Authors:** Suryanarayan Biswal, Janina E. Borgonovo, Carlos L. Freites, Verónica Martínez-Cerdeño, Rajnikant Mishra, Shashank K. Maurya, Estela M. Muñoz

**Affiliations:** ^1^ Department of Human Genetics and Molecular Medicine, Central University of Punjab, Bathinda, India; ^2^ Integrative Biology Program, Institute of Biomedical Sciences, Faculty of Medicine, University of Chile, Santiago, Chile; ^3^ Department of Cell Biology, Physiology and Immunology, Institute of Neurosciences (INc), Autonomous University of Barcelona (UAB), Bellaterra, Spain; ^4^ Department of Pathology and Laboratory Medicine, Institute for Pediatric Regenerative Medicine, Shriners Hospitals for Children of Northern California, and MIND Institute at the UC Davis Medical Center, University of California, Davis School of Medicine, Sacramento, CA, United States; ^5^ Biochemistry and Molecular Biology Laboratory, Department of Zoology, Institute of Science, Banaras Hindu University, Varanasi, India; ^6^ Biochemistry and Molecular Biology Laboratory, Department of Zoology, Faculty of Science, University of Delhi, Delhi, India; ^7^ Institute of Histology and Embryology of Mendoza (IHEM), National University of Cuyo (UNCuyo), National Scientific and Technical Research Council (CONICET), Mendoza, Argentina

**Keywords:** neuroinflammation, neurodegeneration, microglia, t cells, b cells, immunoregulation, meninges

The intricate interplay between the central nervous system (CNS) and the immune system is increasingly recognized as fundamental to both neurological health and disease (1, 2). This second volume of the Community Series in Trends in Neuroimmunology continues to spotlight emerging insights into how brain-resident and peripheral immune cells communicate across anatomical and functional borders, shaping responses in both homeostatic and pathological contexts. Ten peer-reviewed manuscripts, including seven original articles, two reviews, and one opinion, encompass this special volume. Ninety-five authors from research laboratories located in twelve countries: Brazil, Canada, Chile, Denmark, France, Germany, Italy, United Arab Emirates, United Kingdom, United States, Republic of Korea, and Singapore, took part in this initiative. The featured articles collectively deepen our understanding of how immune surveillance, modulation, and dysfunction at CNS interfaces—such as the meninges, choroid plexus, and perivascular spaces—contribute to neurodevelopmental, autoimmune, infectious, and degenerative processes. From the dynamic roles of meningeal immunity and ectopic lymphoid tissues in neuroinflammation, to the surprising effects of peripheral interventions like extracorporeal photopheresis (ECP) or microneedle stimulation in disorders such as stiff person syndrome (SPS) and Parkinson’s disease (PD), respectively, this Research Topic emphasizes the permeability and responsiveness of the brain-immune interface. Novel mechanistic insights into immune cell plasticity, such as interferon-gamma (IFN-γ)–induced homeostatic microglia reprogramming and sustained immunomodulation by cladribine, highlight the therapeutic potential of immune-targeted strategies. Additionally, the exploration of aging-related myelin degradation and immune dysregulation offers promising new directions for understanding cognitive decline. Together, these contributions expand the conceptual framework of neuroimmunology and underscore the translational relevance of immune-CNS cross-talk. As research unravels the bidirectional communication between the brain and the immune system, targeting these interactions may redefine therapeutic landscapes for neurological diseases.

Among the featured contributions, Patel et al. provided a comprehensive review about meningeal immunity in health and disease. The meninges, consisting of three layers (dura mater, arachnoid mater, and pia mater), were once thought to serve primarily as a physical barrier for the CNS. However, recent research has revealed that the meninges play a crucial role in immune function and CNS homeostasis (3). The review discusses details of the anatomy of the meninges and their contribution to immune cell mobilization. It describes the various immune cell populations present in the meninges during steady-state conditions, including T cells, B cells, border-associated macrophages, dendritic cells, neutrophils, mast cells, innate lymphoid cells, and natural killer cells. Each of these cell types has specific functions in maintaining CNS health and responding to pathological conditions (4). The review also discusses the spatial organization of immune cells in the dura mater, particularly the recently discovered dural-associated lymphoid tissues (DALT) (5). The review then explores the role of meningeal immunity in various pathologies, including multiple sclerosis (MS), infections [such as ZIKV (Zika virus), human immunodeficiency virus (HIV), and Toxoplasma gondii], stroke, traumatic brain injury, and neurodegenerative contexts like those in Alzheimer’s disease (AD) and PD. The authors highlighted how meningeal immune responses can contribute to pathology and provide protection in these conditions (6). One of the most intriguing insights from this review is the emerging understanding of the meninges as a dynamic immunological interface rather than a simple physical barrier. The discovery of meningeal lymphatic vessels and organized lymphoid structures within the meninges has challenged previous notions of CNS immune privilege (7). Additionally, the authors also highlighted the connections between meningeal immunity and cognitive function, with certain immune cells and cytokines influencing learning, memory, and social behavior. These suggest a complex interplay between the immune and nervous systems that goes beyond traditional concepts of neuroimmunology. The potential for targeting meningeal immune cells as a novel therapeutic approach for various neurological disorders opens new avenues for research and treatment strategies (8). Overall, the review emphasizes the complex and dynamic nature of meningeal immunity and its significance in both CNS health and disease, highlighting the need for further research to fully understand and potentially manipulate this system for therapeutic purposes.

Tan et al. reviewed the contribution of the brain-border immune niches to the pathogenesis of neurodegenerative disorders. Under non-homeostatic conditions, the immune cells of choroid plexus, meninges, and perivascular spaces can infiltrate the CNS, exacerbating neuroinflammation and neuronal death in pathologies such as AD, PD and MS (9–11). Similarly, cranial bone marrow (CBM) can be a source of lymphoid and myeloid cells for the brain during inflammatory events (12, 13). There is also evidence that obstruction of lymphatic flow delays the clearance of protein aggregates in proteinopathies (14–16). In this review, the authors provided an overview of the different brain-border immune niches, followed by a summary of current knowledge about how their immune cells play a role in the progression of diseases. A special section presented approaches and tools available for monitoring the immune niches such as functional and structural imaging techniques, as well as future research directions in this field (17, 18). Finally, Tan et al. provided a framework for thinking about strategies to target brain-border immune niches in neurological disorders with inflammatory scenarios.

Extracorporeal photopheresis (ECP) is proposed by Castillo-Aleman and Krystkowiak as a potential treatment for SPS, a rare neuroimmunological disorder characterized by progressive muscle rigidity and painful spasms (19). In their opinion article, the authors explained the etiopathophysiology of SPS, which involves autoantibodies targeting various antigens present in inhibitory synapses, particularly anti-GAD65 (glutamic acid decarboxylase) antibodies, and the participation of B cells and GAD65-specific T cells (20). These alter γ-aminobutyric acid (GABA) synthesis and release on synaptic neuronal junctions within the CNS, resulting in impaired neurotransmission and neuronal dysfunction. The authors outlined current therapies for SPS, including symptomatic treatments with diazepam or other benzodiazepines (GABA agonists), and immunotherapies such as corticosteroids, intravenous and subcutaneous immunoglobulins (IVIg and SCIg, respectively), and plasma exchange. They also discussed newer approaches like anti-B cell therapies, autologous anti-CD19 chimeric antigen receptor (CAR) T cells, and hematopoietic stem cell transplantation. However, these treatments have limitations, including heterogeneous clinical responses and potential adverse effects (21). The authors proposed ECP as a rational approach for treating SPS, particularly its classical form. They explained the mechanisms of ECP, which involves exposure of autologous leukocytes to a photosensitizing agent and ultraviolet-A irradiation before reinfusion. This process induces apoptosis in a cell type-dependent manner [T and B cells are more susceptible than monocytes and regulatory T (Treg) cells] and triggers a cascade of immunomodulatory effects (22). Potential benefits of ECP in SPS include its ability to induce tolerance to GAD65-expressing neurons, restore inhibitory signals, and stabilize neuron membranes (23). A unique insight was given by the authors to the investigational use of ECP in SPS, despite the presence of the blood-brain barrier (BBB). They argued that although the BBB may diminish the effects of ECP, the trafficking of immune cells between the periphery and the CNS, and the systemic production of anti-GAD65 antibodies justify exploring ECP as a potential treatment. This perspective challenges the conventional view of the CNS as an immune-privileged site and suggests that peripheral immunomodulation could have significant effects on neurological autoimmune disorders like SPS (24). The authors also proposed a pilot clinical trial (OPTION study; NCT06703333) to evaluate the safety and efficacy of ECP in patients with classical SPS, demonstrating a concrete step towards translating this theoretical approach into clinical practice.

Anti-CD20 monoclonal antibodies (aCD20 mAbs) effectively deplete B cells and are widely used in treating certain forms of MS. Disease progression may persist, suggesting additional mechanisms beyond B cell-mediated pathology (25). The role of B cells in meningeal ectopic lymphoid tissue (mELT) and mELT formation and potential contribution to MS progression even in the absence of B cells, remain unclear (26). Georgieva et al. conducted an original study to compare gene expression profiles of immune cells from cerebrospinal fluid (CSF) and mELT in a spontaneous 2D2xTh EAE (experimental autoimmune encephalomyelitis) murine model of MS. The researchers also applied single-cell RNA sequencing to study the effects of aCD20 mAbs on these compartments. The study found that the immune cell compositions in CSF and mELT were very similar, with both compartments predominantly consisting of B cells and CD4^+^ T lymphocytes. The gene expression profile and pathway enrichment analysis revealed no striking differences between the two compartments. When treated with aCD20 mAbs, the murine CSF showed a complete depletion of B cells, a reduction in naïve CD4^+^ T cells, and a marked increase in macrophages. However, no remarkable differences in regulated genes or pathways were observed between the treated and untreated groups. This study provides several unique insights. Firstly, it suggests that CSF immune cells may serve as a surrogate for studying mELT in EAE, which could potentially be applied to MS research in humans. This is particularly valuable as mELT is not easily accessible in living MS patients (27). Secondly, the observed increase in macrophages in B-cell depleted CSF is a novel finding that warrants further investigation in MS patients treated with aCD20 mAbs. Lastly, the study highlights the complexity of B-cell depletion effects, as it did not significantly alter the inflammatory state of remaining immune cells, despite changing the overall cellular composition. This and other limitations of the study were pointed out by the authors. Nevertheless, their findings contribute to our understanding of the mechanisms underlying MS and the effects of B cell-depleting therapies, potentially informing future treatment strategies for MS and other autoimmune diseases.

Tichauer at al. exposed further evidence on the dynamic balance between pro-inflammatory and anti-inflammatory microglial phenotypes in pathological contexts. The authors investigated the effect of 5-day systemic administration of IFN-γ on infiltrating myeloid cells (MC) and microglial cells (MG) during the peak of EAE, a widely used model of MS (28, 29). IFN-γ treatment markedly decreased the absolute number of CD11b^+^ MC, infiltrated inflammatory cells (CD11b^+^ Ly6G^-^), and demyelination levels within the spinal cord (SC) of EAE mice compared to PBS-treated EAE mice. Moreover, IFN-γ-treated EAE mice displayed a reduced number of activated MC/MG cells alongside an increase in resting MG. Immunofluorescence staining of SC sections from IFN-γ-treated EAE animals, using Iba1 as a microglial marker, revealed cells with a ramified morphology, in contrast to the amoeboid-like shapes observed in control EAE animals. While the authors used the now outdated term ‘resting’ to describe ramified MG, current evidence demonstrates that ramified microglia are highly dynamic, actively extending their processes to continuously monitor and sense the surrounding microenvironment (30–32). The study also explored the regulatory and tolerogenic activity of MG cells induced by IFN-γ. Primary MC/MG cultures from SC of IFN-γ-treated EAE animals, when re-stimulated *ex vivo* with low doses of IFN-γ and myelin oligodendrocyte glycoprotein (MOG_35-55_), showed enhanced induction of CD4^+^ Treg cells and elevated levels of transforming growth factor-beta (TGF-β). In addition, the treated cultures exhibited reduced nitrite production following lipopolysaccharide (LPS) stimulation compared to controls. *In vivo* stimulation of EAE mice with IFN-γ upregulated the expression of the microglial marker CX3CR1 (fractalkine receptor) while downregulating the coinhibitory molecule PD-L1 in MC/MG populations. These MG cells were highly reactive for canonical microglial markers, such as Tmem119, P2ry12, and Hexb, but not for MC, oligodendrocyte, or astrocyte markers, suggesting the presence of a distinct microglial subpopulation (CX3CR1^high^PD-L1^low^ MG). This phenotype was shown to be induced via STAT-1 (signal transducer and activator of transcription 1) signaling, involved in proinflammatory molecules expression (33). Transcriptional profiling of CX3CR1^high^PD-L1^low^ MG cells revealed that *in vivo* IFN-γ stimulation upregulated tolerogenic and anti-inflammatory genes, and downregulated pro-inflammatory genes. Collectively, these findings highlight the dual modulatory effects of IFN-γ on microglial phenotypes and their potential relevance in the context of autoimmune neuroinflammation.

Patients with MS often experience exacerbations of clinical symptoms followed by periods of recovery, known as relapsing-remitting MS (RRMS), where infiltrating blood monocytes play a significant role in neuroinflammation (34). In this context, Rodriguez et al. conducted a comprehensive phenotypic characterization of the monocyte population in MS patients at diagnosis. Peripheral blood mononuclear cells (PBMCs) from early RRMS patients (N=69) were analyzed using mass cytometry, revealing a significant increase in two subsets of CD14^+^ monocytes (Mo) expressing high and intermediate levels of CD206 and CD209 (CD206^high^ CD209^high^ and CD206^int^ CD209^int^, respectively) compared to age- and sex-matched healthy controls (N=29). These molecules are typically expressed in tissue-infiltrating monocyte-derived cells (35–38). Notably, CD206^high^ CD209^high^ Mo exhibited elevated expression of markers such as HLA-DR, CD86, CD45RA, CCR5, CCR2, and CD106 compared to classical monocytes (cMo), indicating an active proinflammatory and migratory phenotype (39–42). The study also found that only 22% of MS patients showed an enrichment of CD206^high^ CD209^high^ Mo, and these individuals presented greater disability than MS patients lacking this subset. Moreover, 75% of patients with CD206^high^ CD209^high^ Mo carried the HLA-DRB1*15:01 allele, a well-known genetic risk factor for MS (43, 44). A second allele, HLA-DQB1*06:02, was also frequent among CD206^high^ CD209^high^ subjects included in this study. Furthermore, single-cell RNA sequencing of CD206^high^ CD209^high^ Mo-like cells isolated from CSF of RRMS patients revealed that they were enriched in monocytic lineage genes, distinct from microglial or macrophage signatures. These CSF-infiltrating cells displayed a characteristic antigen presentation profile and enhanced proinflammatory capacity, suggesting that circulating CD206^high^ CD209^high^ Mo-like cells adopt a pathological phenotype once reaching the CNS. Although the listed limitations of this study, the authors propose that the CD206^high^ CD209^high^ Mo subset may contribute to MS progression, and targeting their polarization, trafficking to the CNS, and antigen presentation process could serve as potential therapeutic strategies.

Holm Hansen et al. contributed to this Research Topic with a case-control exploratory study aimed to evaluate the long-term effects of oral cladribine treatment in patients with MS. There are currently several disease-modifying therapies approved by the US Food and Drug Administration for the RRMS (45–47). Among the high-efficacy therapies, oral administration of cladribine induces temporary immune suppression followed by lymphocyte repopulation and reconstitution of the immune system and its effector functions (48–51). Although cladribine appears to be a therapeutic option, most of the clinical evidence comes from short-term studies. In their article, Holm Hansen et al. presented a longitudinal analysis of the sustained effects of cladribine tablet therapy on the dynamics of circulating immune cell subsets and autoreactivity, during the second year of treatment in patients with RRMS. PBMCs from untreated patients and RRMS patients treated chronically for 52, 60, 72 and 96 weeks (W) were subjected to flow cytometry and FluoroSpot antigen reactivity assay. The authors reported that the therapy has long-term effects and still maintains its ability to modulate immune responses in a cell subset- and time-dependent manner. Mainly, chronic cladribine administration induced significant reductions in circulating memory B cells and proinflammatory B cell responses. It also impaired T and B cell cross-talk, reducing T cell responses to autoantigens possibly presented by B cells, including RAS guanyl releasing protein 2 (RASGRP2), a-B crystallin (CRYAB), myelin basic protein (MBP), and MOG, in a time-dependent fashion. The study by Holm Hansen et al. provides important information for better defining prolonged therapeutic strategies for treating RRMS patients.

Aging is one of the most significant risk factors for cognitive decline, with white matter deterioration playing a crucial role in age-related cognitive impairment, even in the absence of neurodegenerative pathologies like AD (52). Despite evidence linking myelin degeneration to cognitive decline, the interplay between the signals in aging white matter remains unexplored, leaving a critical gap in understanding how microglia-mediated myelin degradation contributes to cognitive impairment. DeVries et al. investigated the role of the immune proteins C1q and CD47 in age-related myelin damage, microglia reactivity, and cognitive decline in rhesus monkeys. These proteins act as ‘eat me’ and ‘don’t eat me’ signals, respectively, regulating in opposition microglial phagocytosis. The study examines changes in C1q and CD47 within the monkey cingulum bundle, a white matter tract in the brain, across normal aging. The researchers used various techniques including immunofluorescence, RNAscope, and ELISA, to analyze the expression and colocalization of C1q and CD47 in relation to MBP and microglia phenotypes. The findings reveal that with age, there is a significant increase in C1q protein in the cingulum bundle, particularly colocalized with myelin. This increase correlated with cognitive impairment in the monkeys. Conversely, CD47 showed a decrease in middle age but paradoxically increased in old age (53). The study also found that microglia become more reactive with age, exhibiting more phagocytic and inflammatory phenotypes. These changes in microglia are associated with increased cognitive impairment (54). A unique insight from this study is the potential role of the balance between ‘eat me’ (C1q) and ‘don’t eat me’ (CD47) signals in age-related myelin damage. The dysregulation of these signals may contribute to an increased vulnerability of myelin to be phagocytized by microglia, potentially leading to cognitive decline (55). This study also highlights the complex and dynamic nature of CD47 expression throughout the aging process, suggesting that its role in myelin maintenance may change at different stages of life. These findings provide a new perspective on the mechanisms underlying age-related cognitive decline and suggest potential targets for therapeutic interventions aimed at preserving cognitive function in aging.

The study by Kim et al. provides insights into how regulation of the peripheral immune system can affect the progression of a neurodegenerative process associated with inflammation. In the search for alternative therapies for PD, acupuncture and moxibustion have emerged as complementary interventions to medications to relieve patients’ motor and non-motor symptoms (56–58). Although it is not known how these types of treatments exert these actions, it has been shown in a PD murine model that acupuncture promotes the clearance of alpha-synuclein aggregates through autophagy (59). Furthermore, acupuncture has a neuromodulatory effect, which is especially relevant in chronic diseases with an inflammatory basis (60–62). Various evidence shows that acupuncture can regulate the function of cellular components of the innate and adaptive immune systems, including mast cells, macrophages, natural killer cells, and CD4^+^ and CD8^+^ lymphocytes (63–66). Kim et al. showed that the attachment of microneedle patches (MPs) to the acupoints GB20 and GB34 in a 6-OHDA (6-hydroxydopamine)-induced PD murine model led to an improvement in the motor and neurodegenerative phenotypes. This improvement was associated with a recovery of the CD4^+^/CD8^+^ ratio at the peripheral and cerebral levels, and a concomitant suppression of both neuroinflammation and damage of dopaminergic neurons in the brain. Therefore, the authors proposed that MPs restore the immune dysfunction in the PD murine model from the periphery to the brain, with significant impact on PD pathology. Despite the limitations of this study listed by the authors, the results open the opportunity to consider acupuncture as a non-invasive complementary therapy for patients with neurological diseases associated with immune alterations, including PD.

Amyotrophic lateral sclerosis (ALS) is a progressive neurological disorder that primarily affects motor neurons. Interestingly, peripheral CD4^+^-expressing Treg cells have been found to inversely correlate with disease progression (67, 68). Zucchi et al. conducted a longitudinal study to evaluate the levels and phenotypes of Tregs in ALS patients, and the potential correlation with ALS progression measured via the ALS functional rating scale (ALSFRS-r) or forced vital capacity (FVC). The study included 21 participants, both men and women, from the placebo arm of the RAP-ALS trial (69, 70). Blood samples were collected at five time points over a period of 54 weeks (8W, 18W, 30W, 42, and 54W). The findings revealed that Treg levels did not exhibit significant changes on average throughout the study. However, specific subpopulations of Tregs may tend to show dynamic alterations: the percentage of PD1^+^ Tregs may decrease, while CD39^+^ Tregs may increase over time. Regarding diverse variables, Zucchi et al. found a positive association between total cholesterol and Tregs, that was particularly evident for CD38^+^ and CXCR3^+^ subsets, suggesting a switch to a more metabolically active profile. Additionally, monocyte counts were positively associated with Treg cell numbers. A modest association and no association between Treg concentrations with FVC and ALSFRS-r declines, respectively, were found in this exploratory study. Due to the lack of significant variation in overall Treg numbers throughout the disease course, the authors cautioned that using Treg numbers alone as a pharmaco-dynamic biomarker for ALS trials may have limited value. Nonetheless, the observed changes in specific Treg phenotypes throughout the ASL course, including PD1^+^ Tregs (71, 72), may merit further investigation for their potential role in ALS progression and therapeutic targeting.

These diverse arrays of articles underscore the intricate and evolving dialogue between the central nervous and immune systems, both at the brain borders and within the parenchyma. The contributions offer cutting-edge insights into how peripheral and brain-resident immune cells influence neurological health, disease progression, and therapeutic responsiveness across conditions like MS, PD, SPS, and age-related cognitive decline. By bridging experimental and clinical perspectives, these studies challenge long-held paradigms of CNS immune privilege and lay critical groundwork for future immunomodulatory strategies in neurodegenerative and neuroinflammatory diseases.
